# Luteinizing hormone supplementation and cumulative live birth rate in assisted reproductive technology cycles among women of advanced reproductive age

**DOI:** 10.3389/fendo.2026.1846492

**Published:** 2026-06-17

**Authors:** Leizhen Xia, Wenting Xia, Lifeng Tian, Jialyu Huang, Houyang Chen, Li Cai, Leixiang Xia, Yan Zhao, Wei Gao

**Affiliations:** 1 Reproductive Medicine Center, Jiangxi Maternal and Child Health Hospital, Nanchang, China; 2 School of Public Health, Jiangxi Medical College, Nanchang University, Nanchang, China; 3 Jiangxi Key Laboratory of Reproductive Health, Jiangxi Maternal and Child Health Hospital, Nanchang, China; 4 Clinical Medical College, Nanchang University, Nanchang, China; 5 Department of Child Health, Jiangxi Maternal and Child Health Hospital, Nanchang, China; 6 Department of Acupuncture, the Affiliated Hospital of Jiangxi University of Traditional Chinese Medicine, Nanchang, China

**Keywords:** advanced reproductive age, assisted reproductive technology, cumulative live birth rate, human menopausal gonadotropin, recombinant luteinizing hormone

## Abstract

**Background:**

The effectiveness of luteinizing hormone (LH) supplementation during assisted reproductive technology (ART) cycles remains controversial, particularly among women of advanced reproductive age. This study aimed to compare the cumulative live birth rate (CLBR) associated with ovarian stimulation using recombinant follicle stimulating hormone (rFSH) alone or in combination with recombinant LH (rLH) or human menopausal gonadotropin (HMG).

**Methods:**

In this retrospective cohort study, 2, 316 women of advanced reproductive age undergoing ART between 2015 and 2024 were included. Cycles were categorized into three groups according to the stimulation regimen: rFSH alone, rFSH+rLH, and rFSH+HMG. Propensity score matching was applied to balance baseline characteristics across three pairwise comparisons. The primary outcome, CLBR, was estimated using cumulative incidence function curves and compared using Fine–Gray models, with permanent failure treated as a competing event.

**Results:**

After propensity score matching, baseline characteristics were well balanced across all comparisons. The rFSH+HMG group required significantly higher total gonadotropin doses and longer stimulation duration than both the rFSH+rLH and rFSH groups (all P < 0.001). Although the number of retrieved oocytes was comparable, the rFSH+rLH group exhibited a higher number of follicles ≥14 mm on trigger day, whereas the rFSH+HMG group had fewer (both P < 0.05). Fresh embryo transfer outcomes did not differ significantly between groups. The rFSH+rLH group showed a higher CLBR compared with the rFSH group (61.31% vs. 51.48%, P = 0.036), while the difference compared with the rFSH+HMG group did not reach statistical significance in unadjusted analyses (59.78% vs. 52.99%, P = 0.102). In multivariable Fine–Gray models, rFSH+rLH was associated with a higher probability of cumulative live birth compared with rFSH alone (adjusted sHR 1.29, 95% CI 1.04–1.61, P = 0.020) and rFSH+HMG (adjusted sHR 1.23, 95% CI 1.01–1.50, P = 0.039).

**Conclusions:**

Among women of advanced reproductive age undergoing ART, recombinant LH supplementation was associated with a higher CLBR compared with rFSH alone or rFSH combined with HMG, whereas the difference versus rFSH combined with HMG was not statistically significant in unadjusted analyses. These findings suggest a potential benefit of rLH, but causality cannot be inferred and results should be interpreted with caution.

## 1 Introduction

Assisted reproductive technology (ART) has become an increasingly important treatment for infertility worldwide (1), particularly as more women delay childbearing to later reproductive ages (2). Women aged ≥35 years, commonly defined as having advanced reproductive age, represent a growing proportion of patients undergoing ART treatment (3). However, reproductive outcomes decline markedly with increasing maternal age. Female fecundity begins to decrease in the early thirties and declines more steeply after the age of 35, largely due to age-related reductions in oocyte quality, diminished ovarian reserve, and endocrine alterations that affect follicular development and oocyte competence (4–6). Consequently, optimizing ovarian stimulation strategies for women of advanced reproductive age has become an important priority in reproductive medicine.

Controlled ovarian stimulation is a crucial component of ART, aiming to recruit multiple follicles in order to increase the number of available oocytes and embryos for transfer. According to the two-cell two-gonadotropin theory, follicle stimulating hormone (FSH) and luteinizing hormone (LH) play complementary roles in follicular development and steroidogenesis through their actions on granulosa and theca cells (7). In routine ART practice, ovarian stimulation is most commonly achieved using recombinant FSH (rFSH) in combination with gonadotropin-releasing hormone (GnRH) agonist or antagonist protocols to prevent premature LH surges (8). However, these suppression protocols may also reduce endogenous LH secretion, potentially affecting follicular steroidogenesis and oocyte development during the stimulation cycle (9, 10).

For this reason, the potential role of exogenous LH supplementation during ovarian stimulation has been widely investigated (11). Biological and clinical evidence suggests that LH supplementation may be particularly beneficial in specific patient populations, including women of advanced reproductive age, individuals with poor or suboptimal ovarian response, or those experiencing profound suppression of endogenous LH during GnRH analogue treatment (12, 13). Age-related reductions in LH bioactivity and androgen production may further contribute to impaired follicular development in older women, providing a biological rationale for LH supplementation in this population (14).

Despite these biological considerations, clinical evidence regarding the effectiveness of LH supplementation in ART remains inconsistent. Some studies and meta-analyses have reported improved implantation or clinical pregnancy rates when LH is added to rFSH in women aged ≥35 years (15–19). In contrast, other studies have failed to demonstrate significant improvements in live birth or other key reproductive outcomes following LH supplementation (20–23). Consequently, the clinical value of routine LH supplementation during ovarian stimulation remains a topic of ongoing debate.

Another important consideration is the source of LH activity used during stimulation. In clinical practice, LH activity can be provided either by recombinant LH (rLH) or by urinary-derived preparations such as human menopausal gonadotropin (HMG), which contains both FSH and LH activity (24). These preparations differ in terms of purity, pharmacokinetic characteristics, and biological activity, which may influence ovarian response and treatment outcomes (25). However, direct comparisons between rLH-based and HMG-based supplementation strategies in women of advanced maternal age remain limited (26).

Furthermore, many previous studies have focused on intermediate outcomes such as the number of retrieved oocytes or pregnancy outcomes following fresh embryo transfer rather than cumulative live birth rate (CLBR) (16, 27). CLBR is increasingly recognized as the most clinically meaningful endpoint in ART, as it reflects the overall reproductive potential derived from a single ovarian stimulation cycle (28, 29). In addition, randomized controlled trials in this field are often limited by relatively small sample sizes and strict eligibility criteria, potentially reducing their generalizability to routine clinical practice (16).

Given the increasing number of women of advanced reproductive age undergoing ART and the ongoing controversy regarding the benefits of LH supplementation, further research is needed to clarify the comparative effectiveness of different gonadotropin combinations in real-world clinical settings. Therefore, the present study aimed to evaluate the effectiveness of different ovarian stimulation strategies involving rFSH alone, rFSH combined with recombinant LH, and rFSH combined with HMG in women of advanced reproductive age undergoing ART treatment, with CLBR as the primary outcome.

## 2 Materials and methods

### 2.1 Study design and population

This retrospective cohort study was conducted at the Reproductive Medicine Center of Jiangxi Provincial Maternal and Child Health Hospital. Women of advanced reproductive age (≥35 years) who underwent ART treatment between January 2015 and December 2024 were screened for eligibility. Clinical and laboratory data were extracted from the institutional electronic medical record database.

**Figure 1:**
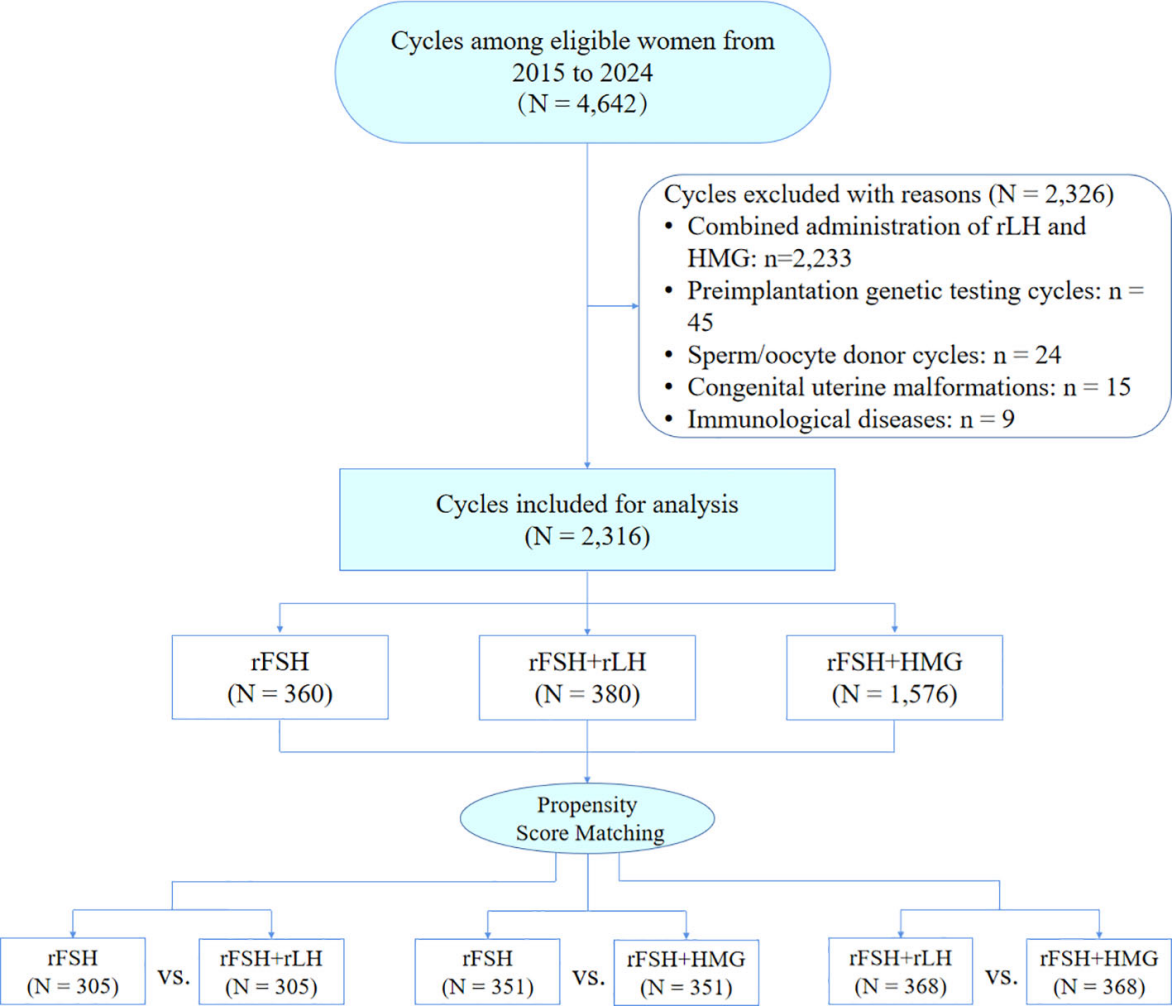
Flowchart of selection for the study.

Eligible cycles included women aged 35 years or older who underwent controlled ovarian stimulation using rFSH within a follicular-phase long-acting GnRH agonist protocol. During the study period, 4, 642 treatment cycles meeting the initial screening criteria were identified. Cycles were excluded if they involved the concurrent administration of rLH and HMG (n = 2, 233), preimplantation genetic testing (n = 45), the use of donor oocytes or donor sperm (n = 24), congenital uterine malformations (n = 15), or immunological disorders (n = 9). After applying these exclusion criteria, 2, 316 treatment cycles were included in the final analysis (**Figure 1**).

Treatment cycles were categorized into three groups according to the ovarian stimulation regimen: rFSH alone, rFSH combined with rLH (rFSH+rLH), or rFSH combined with HMG (rFSH+HMG). In cycles receiving LH activity supplementation, either rLH or HMG was introduced during the mid-follicular phase of ovarian stimulation. The final study population comprised 360 cycles treated with rFSH alone, 380 cycles treated with rFSH+rLH, and 1, 576 cycles treated with rFSH+HMG. Given the observational nature of the study and potential baseline differences among treatment groups, propensity score matching (PSM) was performed to minimize confounding. Three pairwise comparisons were conducted.

The study protocol was reviewed and approved by the Reproductive Medicine Ethics Committee of Jiangxi Maternal and Child Health Hospital (Approval No. SZYX-20260312). The study was conducted in accordance with the principles of the Declaration of Helsinki. All data were anonymized prior to analysis, and the dataset is available from the corresponding author upon reasonable request.

### 2.2 IVF/ICSI treatment procedures

All patients underwent controlled ovarian stimulation using a follicular-phase long-acting GnRH agonist protocol. A long-acting GnRH agonist was administered on day 2 or 3 of the menstrual cycle to achieve pituitary downregulation, including either triptorelin (Diphereline^®^, Beaufour Ipsen, France) or leuprorelin acetate microspheres (Livzon Pharmaceutical Group, China) at a dose of 3.75 mg. Approximately 28 days after GnRH-a administration, patients returned for assessment of pituitary suppression using transvaginal ultrasonography and serum hormone measurements. Downregulation was considered adequate when the endometrial thickness was ≤5 mm, serum FSH was <5 IU/L, LH was <5 IU/L, and estradiol (E2) was <50 pg/mL. After confirmation of pituitary suppression, ovarian stimulation was initiated with rFSH (Gonal-F^®^, Merck Serono, Switzerland). The starting dose of gonadotropins was individualized according to patient characteristics, including age, body mass index (BMI), ovarian reserve markers, and previous treatment history. During stimulation, gonadotropin doses were adjusted based on follicular development monitored by transvaginal ultrasonography, together with serum hormone levels and endometrial thickness.

In selected cycles, LH activity supplementation was generally introduced during the mid-follicular phase (stimulation day 5) when serum LH levels were considered relatively low, follicular growth appeared suboptimal, or clinicians anticipated insufficient endogenous LH support under the profound pituitary suppression induced by the long-acting GnRH agonist protocol. Supplementation was provided as either recombinant LH (rLH; Luveris^®^, Merck Serono, Switzerland) or human menopausal gonadotropin (HMG; Livzon Pharmaceutical Group, China) at a dose of 75 IU/day. However, as this was a retrospective real-world study, no universally predefined biochemical threshold or mandatory protocol dictated the initiation of supplementation; the decision to start LH support and the choice between rLH and HMG were made by treating physicians based on individualized clinical judgment rather than standardized allocation criteria. Factors potentially influencing treatment selection included ovarian response during stimulation, hormonal dynamics, previous clinical history, physician preference, and medication accessibility. In patients receiving HMG supplementation, the concomitant rFSH dose could be adjusted during stimulation according to ovarian response and follicular monitoring, as HMG provides additional FSH activity. These dose adjustments were individualized in routine clinical practice with the aim of maintaining appropriate follicular recruitment while avoiding excessive ovarian stimulation.

Final oocyte maturation was triggered using 250 μg recombinant human chorionic gonadotropin (hCG) (Ovitrelle^®^, Merck Serono, Switzerland) when at least one follicle reached a diameter of ≥19 mm or when at least two follicles reached ≥18 mm. Oocyte retrieval was performed 36–38 hours later under transvaginal ultrasound guidance. Fertilization was achieved using either conventional *in vitro* fertilization (IVF) or intracytoplasmic sperm injection (ICSI), depending on semen parameters. Fertilization was confirmed by the presence of two pronuclei 16–18 hours after insemination. Embryos were cultured at 37 °C in a tri-gas incubator (6% CO₂ and 5% O₂) and evaluated according to standard morphological criteria. When appropriate based on embryo number and quality on day 3, embryos were further cultured to the blastocyst stage. The decision to perform fresh embryo transfer or adopt a freeze-all strategy was made according to endometrial conditions, ovarian response, and other clinical considerations.

Embryos not transferred in the fresh cycle were cryopreserved using vitrification. Frozen–thawed embryo transfers were subsequently performed either in natural cycles or in hormonally prepared endometrial cycles, with or without GnRH agonist pretreatment. One or two embryos were transferred depending on patient characteristics and clinical judgment. Luteal phase support consisted of vaginal progesterone gel (90 mg/day; Crinone^®^, Merck Serono, Switzerland) combined with oral dydrogesterone (20 mg/day; Duphaston^®^, Abbott Biologicals, USA). Luteal support was continued until 10 weeks of gestation in cases of confirmed pregnancy.

### 2.3 Outcome assessment

The primary outcome of this study was the CLBR per ovarian stimulation cycle. CLBR was defined as the probability of achieving at least one live birth from all fresh and subsequent frozen–thawed embryo transfers derived from a single ovarian stimulation cycle, with a follow-up period of at least 2 years (30). Follow-up continued until a live birth occurred or until all embryos obtained from the corresponding stimulation cycle had been transferred without resulting in a live birth.

For the competing risk analysis, each treatment cycle was classified into one of three mutually exclusive outcome states. The event of interest was live birth, defined as the delivery of at least one live-born infant following either a fresh or frozen-thawed embryo transfer. The competing event was permanent failure to achieve a live birth, defined as cycles in which no transferable embryos were obtained or all embryo transfers were completed without resulting in a live birth. Censoring occurred when no live birth was achieved by the end of the follow-up period despite the availability of remaining cryopreserved embryos for future transfer, or when embryo transfers from different stimulation cycles were mixed.

Live birth was defined as the delivery of a live infant at or beyond 22 weeks of gestation (31). Time to live birth was defined as the interval from the initiation of ovarian stimulation to the date of delivery. Ovarian hyperstimulation syndrome (OHSS) was diagnosed according to the Golan classification (32). A biochemical pregnancy was defined as a serum β-hCG level exceeding 20 IU measured 10–12 days after embryo transfer (12 days for cleavage-stage embryos and 10 days for blastocysts). Clinical pregnancy was confirmed by the ultrasonographic detection of one or more intrauterine gestational sacs approximately 30 days after embryo transfer. Multiple pregnancy was defined as the presence of more than one gestational sac or fetal heartbeat detected by ultrasonography. Miscarriage was defined as pregnancy loss occurring before 24 weeks of gestation.

Laboratory outcomes included several embryological indicators. The oocyte retrieval rate was calculated as the proportion of oocytes retrieved from follicles ≥14 mm on the trigger day relative to the total number of oocytes retrieved. The metaphase II (MII) oocyte rate in ICSI cycles was calculated as the number of mature oocytes divided by the total number of retrieved oocytes subjected to ICSI. The normal fertilization rate was defined as the number of two-pronuclear (2PN) zygotes divided by the number of oocytes inseminated. The cleavage rate was calculated as the number of cleaved embryos divided by the number of normally fertilized oocytes. The good-quality embryo rate on day 3 was defined as the proportion of embryos with 7–10 blastomeres, uniform blastomere size, and < 20% fragmentation among normally cleaved embryos. The blastocyst formation rate was defined as the number of embryos developing into viable blastocysts divided by the number of embryos cultured to the blastocyst stage. Transferable embryos were defined as cleavage-stage embryos containing ≥6 blastomeres with <40% fragmentation or blastocysts graded ≥3BB according to the Gardner grading system (33). The cycle cancellation rate was defined as the proportion of treatment cycles in which no transferable embryos were obtained. The fresh embryo transfer rate was calculated as the proportion of cycles in which fresh embryo transfer was performed.

### 2.4 Statistical analysis

All statistical analyses were performed using R software (version 4.5.1). Continuous variables were summarized as mean ± standard deviation (SD) and assessed for normality using the Shapiro–Wilk test. Between-group comparisons were conducted using Student’s t-test or one-way analysis of variance (ANOVA) for normally distributed variables, and the Mann–Whitney U test or Kruskal–Wallis test for non-normally distributed variables, as appropriate. Categorical variables were presented as frequencies and percentages and compared using the χ² test. Missing data for baseline covariates were minimal (<1% for all variables). Therefore, complete-case analysis was performed.

To reduce confounding due to baseline differences between treatment groups, PSM was performed. Propensity scores were estimated using multivariable logistic regression models incorporating the following baseline covariates: age, BMI, duration of infertility, AFC, AMH, basal FSH, basal LH, basal estradiol, basal testosterone, primary infertility, recurrent spontaneous abortion, prior embryo transfer failure, prior cesarean section, intrauterine adhesion, cycle rank, and infertility-related diseases. Matching was performed using a 1:1 nearest-neighbor algorithm without replacement with a caliper width of 0.05. Balance of covariates before and after matching was assessed using absolute standardized mean differences (SMD). An SMD < 0.1 was considered indicative of adequate balance. A pairwise matching strategy was selected instead of simultaneous three-group matching in order to optimize covariate balance within each clinically relevant comparison while preserving the largest possible sample size. Pairwise PSM analyses were conducted independently, and the results should be interpreted as separate comparisons rather than a unified multi-group inference. Propensity score analyses were performed using the R package MatchIt.

Because cumulative live birth may be precluded by permanent treatment failure, competing risk methods were applied. Cumulative incidence functions (CIF) curves were used to estimate the probability of live birth while accounting for the competing event of permanent failure to achieve a live birth. Differences between groups were assessed using Gray’s test. To further evaluate the association between stimulation protocols and CLBR, multivariable Fine–Gray subdistribution hazard models were fitted, adjusting for potential confounders listed in **Table 1**. Results were reported as subdistribution hazard ratios (sHRs) with 95% confidence intervals (CIs). Competing risk analyses were conducted using the R package tidycmprsk. All statistical tests were two-sided, and a P value <0.05 was considered statistically significant.

**Table 1:** Basic characteristics of the study cohort before propensity score matching.

Variables	rFSH (n = 360)	rFSH+rLH (n = 380)	rFSH+HMG (n = 1576)	P-value
Age (years)	36.89 ± 1.94	36.87 ± 2.02	37.30 ± 2.18	< 0.001
BMI (kg/m2)	22.28 ± 2.94	22.05 ± 2.81	22.33 ± 3.00	0.342
Duration of infertility (years)	4.80 ± 4.15	4.89 ± 3.90	5.13 ± 4.24	0.283
AFC	13.43 ± 5.34	14.07 ± 5.75	11.74 ± 5.15	< 0.001
AMH (ng/mL)	3.90 ± 2.71	4.01 ± 2.64	3.40 ± 3.48	< 0.001
Basal FSH (IU/L)	6.02 ± 2.30	5.95 ± 1.94	6.60 ± 2.27	< 0.001
Basal LH (IU/L)	5.43 ± 3.79	5.30 ± 3.3	5.25 ± 4.05	0.284
Basal estradiol (pg/mL)	50.87 ± 43.66	49.63 ± 44.63	47.03 ± 41.53	0.464
Basal testosterone (ng/dL)	26.91 ± 17.27	28.29 ± 18.44	27.99 ± 17.52	0.527
Primary Infertility, n (%)	53 (14.72)	69 (18.16)	289 (18.34)	0.262
RSA, n (%)	32 (8.89)	32 (8.42)	170 (10.79)	0.275
Prior ET failure, n (%)	57 (15.83)	54 (14.21)	230 (14.59)	0.797
Prior cesarean section, n (%)	95 (26.39)	108 (28.42)	362 (22.97)	0.054
Intrauterine adhesion, n (%)	90 (25.00)	70 (18.42)	266 (16.88)	0.002
Rank of cycle, n (%)				0.897
1	284 (78.89)	306 (80.53)	1275 (80.9)	
2	61 (16.94)	60 (15.79)	236 (14.97)	
≥3	15 (4.17)	14 (3.68)	65 (4.12)	
Infertility diseases, n (%)				
Tubal factor	240 (66.67)	263 (69.21)	1104 (70.05)	0.452
Male factor	106 (29.44)	100 (26.32)	418 (26.52)	0.506
Endometriosis	13 (3.61)	19 (5.00)	79 (5.01)	0.521
Ovulatory dysfunction	29 (8.06)	39 (10.26)	117 (7.42)	0.186
Diminished Ovarian Reserve	16 (4.44)	18 (4.74)	129 (8.19)	0.007
Uterine factor	52 (14.44)	51 (13.42)	219 (13.90)	0.922
Unexplained	28 (7.78)	25 (6.58)	83 (5.27)	0.153

Data are presented as mean ± SD or number (percentage). Abbreviations: *rFSH*, recombinant follicle stimulating hormone; *rLH*, recombinant luteinizing hormone; *HMG*, human menopausal gonadotropin; *BMI*, body mass index; *AFC*, antral follicle count; *AMH*, anti-Müllerian hormone; *RSA*, recurrent spontaneous abortion; *ET*, embryo transfer.

## 3 Results

### 3.1 Baseline characteristics before and after PSM

**Figure 2:**
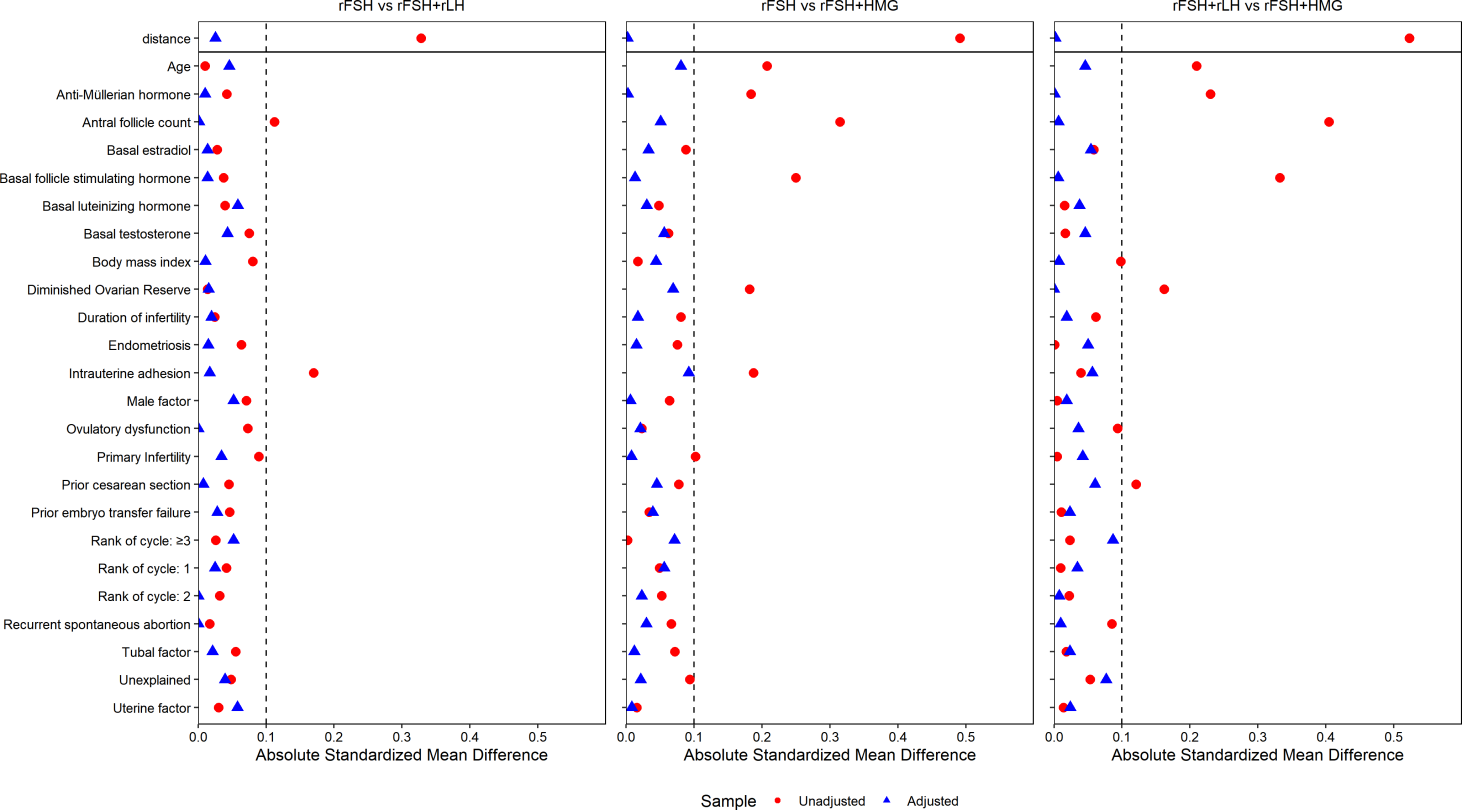
Love plot of absolute standardized mean differences before and after propensity score matching for the three pairwise comparisons.

Prior to PSM, significant differences were observed between groups in age, antral follicle count, anti-Müllerian hormone, basal FSH, and the prevalence of diminished ovarian reserve and intrauterine adhesions (all P < 0.05). The rFSH+HMG group was characterized by older age and poorer ovarian reserve compared with the other groups (**Table 1**). After matching, all absolute standardized mean differences were below 0.1, demonstrating adequate covariate balance (**Figure 2**), and the baseline characteristics showed no significant differences across any of the three pairwise comparisons (all P > 0.05) (**Table 2**).

**Table 2:** Basic characteristics of the study cohort after propensity score matching.

Variables	rFSH (n = 305)	rFSH+rLH (n = 305)	P-value	rFSH (n = 351)	rFSH+HMG (n = 351)	P-value	rFSH+rLH (n = 368)	rFSH+HMG (n = 368)	P-value
Age (years)	36.89 ± 1.86	36.8 ± 1.92	0.359	36.92 ± 1.95	36.77 ± 1.75	0.526	36.9 ± 2.02	36.81 ± 1.87	0.822
BMI (kg/m2)	22.17 ± 2.65	22.14 ± 2.86	0.686	22.31 ± 2.96	22.18 ± 2.97	0.322	22.1 ± 2.82	22.12 ± 2.76	0.880
Duration of infertility (years)	4.92 ± 4.27	4.85 ± 3.89	0.600	4.83 ± 4.18	4.76 ± 4.00	0.818	4.87 ± 3.92	4.79 ± 4.08	0.436
AFC	13.64 ± 5.12	13.64 ± 5.61	0.820	13.27 ± 5.28	13.54 ± 5.44	0.482	13.71 ± 5.4	13.74 ± 5.37	0.853
AMH (ng/mL)	3.92 ± 2.77	3.89 ± 2.60	0.761	3.83 ± 2.68	3.84 ± 2.71	0.960	3.9 ± 2.59	3.9 ± 2.89	0.338
Basal FSH (IU/L)	5.94 ± 2.17	5.97 ± 1.88	0.657	6.08 ± 2.29	6.11 ± 2.03	0.311	5.99 ± 1.96	6.00 ± 1.73	0.397
Basal LH (IU/L)	5.25 ± 3.51	5.44 ± 3.34	0.423	5.39 ± 3.72	5.50 ± 3.72	0.519	5.23 ± 3.21	5.11 ± 2.96	0.763
Basal estradiol (pg/mL)	50.8 ± 43.54	50.19 ± 45.55	0.816	50.27 ± 41.94	51.70 ± 53.02	0.420	49.49 ± 44.00	47.07 ± 46.81	0.209
Basal testosterone (ng/dL)	27.13 ± 16.27	27.93 ± 18.93	0.873	27.02 ± 17.35	27.99 ± 17.03	0.379	28.11 ± 18.53	28.95 ± 15.97	0.256
Primary Infertility, n (%)	49 (16.07)	53 (17.38)	0.745	52 (14.81)	51 (14.53)	1.000	68 (18.48)	62 (16.85)	0.629
RSA, n (%)	24 (7.87)	24 (7.87)	1.000	31 (8.83)	34 (9.69)	0.795	32 (8.70)	31 (8.42)	1.000
Prior ET failure, n (%)	47 (15.41)	44 (14.43)	0.820	53 (15.1)	48 (13.68)	0.667	51 (13.86)	54 (14.67)	0.833
Prior cesarean section, n (%)	81 (26.56)	82 (26.89)	1.000	93 (26.5)	100 (28.49)	0.612	100 (27.17)	110 (29.89)	0.463
Intrauterine adhesion, n (%)	59 (19.34)	61 (20.00)	0.919	83 (23.65)	69 (19.66)	0.234	68 (18.48)	60 (16.30)	0.496
Rank of cycle, n (%)			0.800			0.550			0.430
1	242 (79.34)	245 (80.33)		279 (79.49)	287 (81.77)		298 (80.98)	303 (82.34)	
2	51 (16.72)	51 (16.72)		57 (16.24)	54 (15.38)		56 (15.22)	57 (15.49)	
≥3	12 (3.93)	9 (2.95)		15 (4.27)	10 (2.85)		14 (3.80)	8 (2.17)	
Infertility diseases, n (%)									
Tubal factor	213 (69.84)	210 (68.85)	0.861	238 (67.81)	236 (67.24)	0.936	254 (69.02)	258 (70.11)	0.810
Male factor	88 (28.85)	81 (26.56)	0.587	102 (29.06)	103 (29.34)	1.000	98 (26.63)	101 (27.45)	0.868
Endometriosis	11 (3.61)	10 (3.28)	1.000	13 (3.70)	12 (3.42)	1.000	18 (4.89)	14 (3.80)	0.588
Ovulatory dysfunction	26 (8.52)	26 (8.52)	1.000	29 (8.26)	31 (8.83)	0.893	35 (9.51)	31 (8.42)	0.699
Diminished Ovarian Reserve	15 (4.92)	16 (5.25)	1.000	16 (4.56)	11 (3.13)	0.432	18 (4.89)	18 (4.89)	1.000
Uterine factor	44 (14.43)	38 (12.46)	0.553	51 (14.53)	52 (14.81)	1.000	51 (13.86)	54 (14.67)	0.833
Unexplained	20 (6.56)	23 (7.54)	0.752	25 (7.12)	27 (7.69)	0.885	24 (6.52)	17 (4.62)	0.335

Data are presented as mean ± SD or number (percentage). Abbreviations: *rFSH*, recombinant follicle stimulating hormone; *rLH*, recombinant luteinizing hormone; *HMG*, human menopausal gonadotropin; *BMI*, body mass index; *AFC*, antral follicle count; *AMH*, anti-Müllerian hormone; *RSA*, recurrent spontaneous abortion; *ET*, embryo transfer.

### 3.2 Ovulation stimulation and embryo culture outcomes

After PSM, several differences in ovarian stimulation and embryological outcomes were observed between groups (**Tables 3**, **4**). Although the starting dose of gonadotropins was comparable across groups, the total gonadotropin dose and duration of stimulation were significantly higher in the rFSH+HMG group than in the rFSH+rLH and rFSH groups (all P < 0.001). While the number of oocytes retrieved was similar across groups, the rFSH+rLH group had the highest number of follicles ≥14 mm on the trigger day, whereas the rFSH+HMG group had the fewest (both P < 0.05). In terms of embryo culture outcomes, the rFSH+HMG group demonstrated a lower blastocyst formation rate than both comparator groups (both P < 0.05). Consequently, the rFSH+rLH group produced a higher number of transferable embryos and exhibited a significantly lower cycle cancellation rate than the rFSH group (P < 0.001).

**Table 3:** Ovarian stimulation characteristics of the study cohort after propensity score matching.

Variables	rFSH (n = 305)	rFSH+rLH (n = 305)	P-value	rFSH (n = 351)	rFSH+HMG (n = 351)	P-value	rFSH+rLH (n = 368)	rFSH+HMG (n = 368)	P-value
Gn starting dose (IU)	163.25 ± 61.74	158.87 ± 58.49	0.237	164.18 ± 61.82	159.88 ± 63.84	0.061	157.73 ± 57.36	159.50 ± 66.21	0.400
Gn total dose (IU)	1594.02 ± 623.29	1641.75 ± 541.63	0.129	1618.31 ± 620.17	2300.11 ± 870.67	< 0.001	1620.77 ± 539.77	2282.93 ± 831.12	< 0.001
Gn duration (days)	9.48 ± 1.76	9.98 ± 1.40	0.001	9.54 ± 1.76	11.45 ± 2.53	< 0.001	9.93 ± 1.43	11.38 ± 2.33	< 0.001
Estradiol on trigger day (pg/mL)	2300.91 ± 1270.13	2056.91 ± 1096.59	0.010	2304.26 ± 1264.97	2294.36 ± 1293.61	0.695	2032.70 ± 1118.67	2402.85 ± 1461.26	< 0.001
LH level on trigger day (IU/L)	1.32 ± 1.67	1.28 ± 0.65	0.131	1.42 ± 1.55	1.23 ± 0.85	0.201	1.24 ± 0.62	1.29 ± 1.16	0.128
Progestin on trigger day (ng/mL)	0.74 ± 0.43	0.63 ± 0.43	< 0.001	0.74 ± 0.42	0.78 ± 0.42	0.115	0.61 ± 0.41	0.81 ± 0.41	< 0.001
Endometrial thickness on trigger day (mm)	10.93 ± 2.81	11.00 ± 2.8	0.611	10.85 ± 2.83	11.01 ± 2.98	0.451	10.98 ± 2.80	10.79 ± 2.69	0.553
Number of follicles ≥ 14 mm on trigger day	9.72 ± 4.21	10.56 ± 3.83	0.028	9.69 ± 4.20	8.85 ± 3.61	0.001	10.44 ± 3.75	8.88 ± 3.54	< 0.001
No. of oocytes retrieved	12.99 ± 6.42	13.44 ± 5.86	0.413	12.79 ± 6.26	12.46 ± 6.39	0.220	13.25 ± 5.77	12.78 ± 6.71	0.062
Oocyte retrieval rate (%)	136.45 ± 49.43	128.80 ± 38.74	0.161	135.14 ± 47.50	144.60 ± 59.9	0.016	128.82 ± 38.91	146.66 ± 59.68	< 0.001
Moderate-to-severe OHSS, n (%)	8 (2.62)	4 (1.31)	0.382	11 (3.13)	5 (1.42)	0.206	6 (1.63)	7 (1.90)	1.000

Data are presented as mean ± SD or number (percentage). Abbreviations: *rFSH*, recombinant follicle stimulating hormone; *rLH*, recombinant luteinizing hormone; *HMG*, human menopausal gonadotropin; *Gn*, gonadotrophin; *LH*, luteinizing hormone; *OHSS*, ovarian hyperstimulation syndrome.

**Table 4:** Embryo culture outcomes of the study cohort after propensity score matching.

Variables	rFSH (n = 305)	rFSH+rLH (n = 305)	P-value	rFSH (n = 351)	rFSH+HMG (n = 351)	P-value	rFSH+rLH (n = 368)	rFSH+HMG (n = 368)	P-value
Fertilization method, n (%)			0.003			0.133			0.133
Cancellation of fertilization	15 (4.92)	1 (0.33)		17 (4.84)	7 (1.99)		1 (0.27)	7 (1.9)	
IVF	238 (78.03)	239 (78.36)		267 (76.07)	264 (75.21)		287 (77.99)	292 (79.35)	
ICSI	43 (14.1)	52 (17.05)		55 (15.67)	63 (17.95)		63 (17.12)	52 (14.13)	
IVF+ICSI	9 (2.95)	13 (4.26)		12 (3.42)	17 (4.84)		17 (4.62)	17 (4.62)	
No. of MII oocytes for ICSI	9.86 ± 4.48	9.73 ± 4.33	0.994	9.91 ± 4.33	9.49 ± 4.2	0.675	9.57 ± 4.56	10.4 ± 5.52	0.583
MII oocytes rate for ICSI	74.2 ± 14.73	72.1 ± 16.39	0.517	75 ± 15.44	76.49 ± 13.85	0.795	73.78 ± 16.22	75.85 ± 16.61	0.389
No. of normally fertilized oocytes	8.01 ± 4.74	8.05 ± 4.33	0.784	7.87 ± 4.61	7.58 ± 4.57	0.299	8 ± 4.23	7.99 ± 4.71	0.539
Normally fertilized rate (%)	65.41 ± 18.94	64.87 ± 19.13	0.534	65.43 ± 19.03	64.53 ± 22.31	0.791	65.09 ± 19.44	65.99 ± 21.64	0.355
No. of cleaved embryos	7.71 ± 4.61	7.81 ± 4.29	0.669	7.6 ± 4.51	7.31 ± 4.5	0.310	7.75 ± 4.2	7.73 ± 4.61	0.554
Cleaved rate (%)	96.09 ± 10.05	96.73 ± 9.49	0.281	96.31 ± 9.67	95.77 ± 9.7	0.661	96.49 ± 9.62	96.58 ± 8.42	0.725
No. of good-quality embryos	2.43 ± 2.29	2.14 ± 1.92	0.296	2.36 ± 2.22	2.21 ± 2.18	0.353	2.2 ± 1.92	2.42 ± 2.27	0.420
Good-quality embryos rate (%)	31.15 ± 23.4	28.81 ± 24.03	0.163	31.16 ± 23.28	30.12 ± 25.05	0.314	29.55 ± 24.04	30.95 ± 23.22	0.360
No. of blastocysts	2.18 ± 2.61	2.25 ± 2.27	0.198	2.08 ± 2.55	1.57 ± 2.12	0.015	2.19 ± 2.32	1.67 ± 2.13	< 0.001
Blastocyst formation rate (%)	40.59 ± 32.08	43.72 ± 30.34	0.231	39.48 ± 31.87	34.32 ± 32.07	0.034	42.7 ± 30.77	35.12 ± 30.93	0.001
No. of transferable embryos	3.68 ± 2.47	3.84 ± 2.14	0.209	3.6 ± 2.4	3.29 ± 2.19	0.064	3.84 ± 2.17	3.51 ± 2.22	0.022
Oocyte utilization rate (%)	30.11 ± 16.57	30.92 ± 15.28	0.406	29.81 ± 16.22	28.16 ± 16.15	0.186	31.13 ± 15.15	29.36 ± 16.44	0.051
Cycle cancellation rate, n (%)	27 (8.85)	7 (2.3)	< 0.001	29 (8.26)	22 (6.27)	0.383	9 (2.45)	19 (5.16)	0.083
Fresh embryo transfer rate, n (%)	220 (72.13)	254 (83.28)	0.001	255 (72.65)	275 (78.35)	0.095	304 (82.61)	292 (79.35)	0.302

Data are presented as mean ± SD or number (percentage). Abbreviations: *rFSH*, recombinant follicle stimulating hormone; *rLH*, recombinant luteinizing hormone; *HMG*, human menopausal gonadotropin; IVF, *in vitro* fertilization; ICSI, intracytoplasmic sperm injection; MII, metaphase II.

### 3.3 Pregnancy outcomes of fresh embryo transfer cycles and CLBR

Analysis of fresh embryo transfer cycles showed no significant differences among groups in the rates of biochemical pregnancy, clinical pregnancy, miscarriage, or live birth (all P > 0.05) (**Table 5**). In contrast, differences emerged in cumulative pregnancy outcomes. The rFSH+rLH group achieved a significantly higher CLBR compared with the rFSH group (61.31% vs. 51.48%, P = 0.036), whereas the difference versus the rFSH+HMG group was not statistically significant (59.78% vs. 52.99%, P = 0.102). Correspondingly, the permanent failure rate was lowest in the rFSH+rLH group (28.2%) and highest in the rFSH group (33.11%).

**Table 5:** Fresh embryo transfer and cumulative pregnancy outcomes of the study cohort after propensity score matching.

Variables	rFSH (n = 305)	rFSH+rLH (n = 305)	P-value	rFSH (n = 351)	rFSH+HMG (n = 351)	P-value	rFSH+rLH (n = 368)	rFSH+HMG (n = 368)	P-value
Fresh transfer outcomes									
Single embryo transfer, n (%)	91 (41.36)	107 (42.13)	0.941	101 (39.61)	88 (32)	0.083	121 (39.8)	95 (32.53)	0.078
Blastocyst transfer, n (%)	70 (31.82)	78 (30.71)	0.872	77 (30.2)	57 (20.73)	0.016	89 (29.28)	55 (18.84)	0.004
Biochemical pregnancy, n (%)	144 (65.45)	163 (64.17)	0.846	165 (64.71)	172 (62.55)	0.670	196 (64.47)	200 (68.49)	0.341
Clinical pregnancy, n (%)	124 (56.36)	143 (56.3)	1.000	141 (55.29)	157 (57.09)	0.742	173 (56.91)	179 (61.3)	0.314
Multiple pregnancy, n (%)	23 (18.55)	29 (20.28)	0.840	25 (17.73)	39 (24.84)	0.177	37 (21.39)	43 (24.02)	0.644
Miscarriage, n (%)	31 (25)	34 (23.78)	0.929	33 (23.4)	31 (19.75)	0.531	41 (23.7)	43 (24.02)	1.000
Live birth, n (%)	92 (41.82)	107 (42.13)	1.000	107 (41.96)	123 (44.73)	0.579	130 (42.76)	134 (45.89)	0.493
Cumulative pregnancy outcomes			0.036			0.117			0.102
Live birth	157 (51.48)	187 (61.31)		176 (50.14)	183 (52.14)		220 (59.78)	195 (52.99)	
Permanent failure	101 (33.11)	86 (28.2)		122 (34.76)	133 (37.89)		108 (29.35)	135 (36.68)	
Censored	47 (15.41)	32 (10.49)		53 (15.1)	35 (9.97)		40 (10.87)	38 (10.33)	
The time to live birth (days)	393.29 ± 227.82	409.4 ± 283.24	0.383	389.8 ± 219.75	365.58 ± 137.36	0.689	399.08 ± 263.96	371 ± 160.5	0.296

Data are presented as mean ± SD or number (percentage). Abbreviations: *rFSH*, recombinant follicle stimulating hormone; *rLH*, recombinant luteinizing hormone; *HMG*, human menopausal gonadotropin.

**Figure 3:**
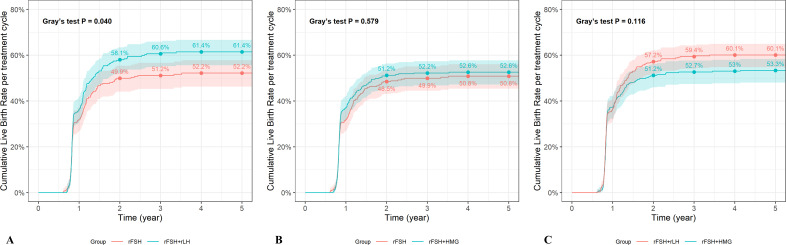
Cumulative incidence function curves for cumulative live birth after propensity score matching. **(A)** rFSH vs. rFSH+rLH. **(B)** rFSH vs. rFSH+HMG. **(C)** rFSH+rLH vs. rFSH+HMG. Shaded areas represent 95% confidence intervals.

As shown in **Figure 3**, CIF curves suggested a trend toward higher CLBR over time in the rFSH+rLH group compared with the rFSH and rFSH+HMG groups (Gray’s test: P = 0.040 and P = 0.116).

### 3.4 Competing risk analysis for CLBRs

In multivariable Fine–Gray models adjusting for potential confounders, rLH supplementation remained significantly associated with a higher CLBR (**Table 6**). Compared with rFSH alone, the rFSH+rLH group had a significantly higher CLBR (sHR 1.29, 95% CI 1.04–1.61, P = 0.020). Similarly, rFSH+rLH also showed a significant advantage over rFSH+HMG (sHR 1.23, 95% CI 1.01–1.50, P = 0.039). In contrast, no significant difference in CLBR was observed between the rFSH+HMG and rFSH groups (sHR 1.04, 95% CI 0.84–1.28, P = 0.720). Several covariates were independently associated with CLBR, including age, prior cesarean section, intrauterine adhesion, and endometriosis.

**Table 6:** Competing risk models for cumulative live birth rates.

Factors	sHR (95% CI)	P-value	sHR (95% CI)	P-value	sHR (95% CI)	P-value
Group	1.29 (1.04-1.61)a	0.020	1.04 (0.84-1.28)b	0.720	1.23 (1.01-1.50)c	0.039
Age	0.86 (0.81-0.92)	<0.001	0.86 (0.80-0.92)	<0.001	0.87 (0.82-0.92)	<0.001
BMI	1.02 (0.98-1.06)	0.360	1.02 (0.99-1.06)	0.260	1.00 (0.96-1.03)	0.910
Duration of infertility	0.97 (0.94-1.00)	0.051	1.00 (0.97-1.03)	0.940	0.98 (0.95-1.00)	0.110
AFC	1.01 (0.98-1.04)	0.400	1.01 (0.99-1.04)	0.300	1.01 (0.99-1.04)	0.260
AMH	1.03 (0.98-1.08)	0.310	0.98 (0.94-1.03)	0.400	0.99 (0.95-1.03)	0.650
Basal FSH	0.94 (0.87-1.01)	0.095	0.97 (0.91-1.04)	0.350	0.92 (0.85-0.99)	0.020
Basal LH	1.02 (0.98-1.05)	0.300	1.03 (1.01-1.06)	0.020	1.04 (1.00-1.08)	0.026
Basal estradiol	1.00 (1.00-1.00)	0.460	1.00 (0.99-1.00)	0.016	1.00 (1.00-1.00)	0.420
Basal testosterone	1.00 (0.99-1.01)	0.620	1.00 (0.99-1.01)	0.870	1.00 (0.99-1.01)	0.790
Primary Infertility, yes vs. no	1.05 (0.77-1.42)	0.760	1.11 (0.80-1.54)	0.520	1.10 (0.84-1.45)	0.490
RSA, yes vs. no	0.72 (0.48-1.10)	0.130	1.03 (0.69-1.53)	0.890	0.88 (0.60-1.29)	0.500
Prior ET failure, yes vs. no	1.41 (0.79-2.52)	0.240	0.94 (0.54-1.65)	0.840	1.92 (1.04-3.55)	0.037
Prior cesarean section, yes vs. no	0.68 (0.52-0.90)	0.007	0.76 (0.59-0.99)	0.042	1.00 (0.80-1.26)	0.980
Intrauterine adhesion, yes vs. no	0.64 (0.48-0.86)	0.003	0.81 (0.62-1.06)	0.130	0.72 (0.55-0.96)	0.024
Rank of cycle, 2 vs. 1	0.70 (0.40-1.22)	0.210	0.94 (0.57-1.53)	0.790	0.44 (0.24-0.77)	0.005
Rank of cycle, ≥3 vs. 1	1.24 (0.62-2.46)	0.540	0.81 (0.36-1.81)	0.600	0.58 (0.23-1.50)	0.270
Tubal factor, yes vs. no	0.94 (0.71-1.24)	0.660	1.00 (0.76-1.33)	0.980	0.83 (0.64-1.07)	0.150
Male factor, yes vs. no	0.97 (0.76-1.24)	0.820	0.85 (0.66-1.10)	0.230	0.99 (0.80-1.24)	0.960
Endometriosis, yes vs. no	1.97 (1.31-2.97)	0.001	1.40 (0.84-2.31)	0.190	1.54 (0.99-2.38)	0.054
Ovulatory dysfunction, yes vs. no	0.98 (0.65-1.48)	0.920	1.00 (0.67-1.49)	0.990	0.94 (0.64-1.38)	0.750
Diminished ovarian reserve, yes vs. no	0.90 (0.49-1.64)	0.730	0.97 (0.50-1.89)	0.930	0.95 (0.53-1.69)	0.860
Uterine factor, yes vs. no	0.88 (0.63-1.23)	0.470	0.74 (0.55-1.01)	0.058	0.79 (0.58-1.09)	0.150
Unexplained, yes vs. no	1.22 (0.73-2.05)	0.450	1.17 (0.75-1.82)	0.490	0.94 (0.58-1.52)	0.800

^a^ rFSH+rLH vs. rFSH.

^b^ rFSH+HMG vs. rFSH.

^c^ rFSH+rLH vs. rFSH+HMG.

*FSH*, follicle stimulating hormone; *LH*, luteinizing hormone; *HMG*, human menopausal gonadotropin; *BMI*, body mass index; *AFC*, antral follicle count; *AMH*, anti-Müllerian hormone; *RSA*, recurrent spontaneous abortion; *ET*, embryo transfer.

## 4 Discussion

In this retrospective cohort study of women with advanced reproductive age undergoing ART, supplementation with rLH during ovarian stimulation was associated with a modest increase in CLBR compared with rFSH alone. Although adjusted competing-risk analyses also suggested a possible association when compared with rFSH+HMG, the unadjusted comparison did not reach statistical significance. Importantly, fresh embryo transfer outcomes were similar across stimulation protocols, suggesting that the potential benefit of rLH supplementation may be more apparent in cumulative reproductive outcomes than in fresh-cycle success.

An important feature of the present study is the use of CLBR as the primary endpoint. Prior investigations into LH supplementation have largely centered on intermediate endpoints, including trigger-day estradiol levels (22), the number of retrieved oocytes (27), and pregnancy outcomes following fresh embryo transfer (17–21, 34, 35). In contrast, CLBR reflects the overall reproductive potential derived from a single ovarian stimulation cycle and is increasingly regarded as the most clinically relevant outcome in ART. This is particularly significant when comparing stimulation protocols, as the protocol dictates oocyte yield and embryo availability, which are direct determinants of CLBR (36).

Our findings showed that rLH supplementation improved CLBR despite having no significant effect on fresh-cycle outcomes. This pattern suggests that the clinical benefit of rLH may be mediated through improved embryo availability rather than enhanced implantation probability. Similar observations have been reported in a large real-world study from Germany (37). Evidence from most randomized trials in women of advanced reproductive age has also shown no significant differences in pregnancy outcomes following fresh embryo transfer (20–23). For example, A multicenter randomized controlled trial conducted in the Netherlands demonstrated supplementation of LH during the second half of the follicular phase has no effect on ovarian response or on pregnancy rates in women of 35 years and older undergoing GnRH antagonist IVF/ICSI cycles (21).

However, some studies have suggested that the effect of LH supplementation may vary according to age subgroups. In a randomized trial by Bosch et al., although no overall benefit was observed among all advanced-age women, subgroup analysis revealed that women aged 36–39 years receiving rLH supplementation had a significantly higher implantation rate (OR 1.56, 95% CI 1.04–2.33) (19). Similarly, a recent meta-analysis of randomized trials reported that rFSH combined with rLH significantly improved implantation and clinical pregnancy rates compared with rFSH alone in women aged 35–40 years (16).

The observation that rLH supplementation increased the number of follicles ≥14 mm on the trigger day in the present study is consistent with the concept that LH promotes the maturation of dominant follicles. Previous studies have suggested that LH activity may suppress the growth of small follicles while favoring the maturation of larger follicles during ovarian stimulation (38). Such selective follicular maturation may improve the developmental competence of retrieved oocytes, potentially leading to higher embryo quality and greater cumulative reproductive success.

Notably, the present study demonstrated a reduced cycle cancellation rate in the rFSH+rLH group. Avoiding cancellation due to a lack of transferable embryos is crucial, especially for women of advanced reproductive age with compromised embryo potential. While previous studies have prioritized pregnancy outcomes, fewer have examined this endpoint directly. Evidence indicates that LH supplementation improves oocyte competence and embryonic development through androgen synthesis and granulosa cell modulation (7, 9). Specifically, LH promotes thecal androgen production and enhances FSH receptor expression, facilitating follicular maturation (9). These mechanisms likely account for the increased yield of transferable embryos and the reduced cancellation rate in the rLH cohort.

Variations in stimulation protocols may underlie the heterogeneity of findings across studies. Most studies investigating LH supplementation in women of advanced reproductive age have utilized GnRH antagonist or conventional long GnRH agonist protocol (20–22, 26, 37). In contrast, the present study adopted a follicular-phase long-acting GnRH agonist protocol. This protocol is widely implemented in China due to its reported benefits in improving endometrial receptivity (39–41). However, this protocol induces profound pituitary suppression, resulting in significantly diminished endogenous LH secretion during ovarian stimulation (42, 43). Under such conditions, exogenous LH supplementation may exert a more pronounced physiological impact. To our knowledge, studies specifically assessing LH supplementation within this long-acting protocol in advanced-age women are limited, highlighting the clinical relevance of our findings.

The timing of LH supplementation varies significantly across studies and may influence outcomes. While several randomized trials initiated rLH at the start of ovarian stimulation (19, 44), others introduced it during the late follicular phase (20, 21) or concurrent with GnRH antagonist administration (22). In the present study, LH supplementation was initiated during the mid-follicular phase, coinciding with the emergence of LH receptors on granulosa cells (45). Physiologically, LH plays a pivotal role in the mid-follicular phase by supporting steroidogenesis, follicular maturation, and oocyte competence (10). Consequently, this timing represents a physiologically sound strategy that promotes follicular maturation while minimizing excessive early follicular recruitment. Furthermore, compared with continuous supplementation, the mid-cycle addition strategy reduces medication costs, thereby improving treatment accessibility for patients with financial constraints.

This study also compared rLH supplementation with HMG. Although adjusted analyses suggested a higher CLBR with the rFSH+rLH protocol versus rFSH+HMG, the non-significant unadjusted comparison warrants caution. Thus, the robustness and real-world relevance of this potential benefit remain uncertain, particularly given considerations of treatment cost, medication burden, and patient selection. Differences in embryo transfer strategy may also have influenced cumulative outcomes. Despite comparable fresh transfer pregnancy outcomes, the rFSH+HMG group had lower rates of single embryo transfer and blastocyst transfer. Since single blastocyst transfers are generally indicative of better embryo quantity and quality, these differences may indirectly reflect variations in developmental potential and could have contributed to the observed disparity in CLBR. Direct comparisons between rLH and HMG in women of advanced reproductive age remain scarce. While a small samples study indicated potentially higher pregnancy rates with rLH, the difference was not statistically significant (26). Nevertheless, accumulating evidence suggests that recombinant LH offers distinct advantages in specific clinical contexts, including improved follicular synchronization, enhanced steroidogenic activity, and potentially superior embryo developmental competence (46, 47).

Although both preparations provide LH activity, recombinant LH and HMG are not biologically equivalent and differ substantially in composition, pharmacokinetic properties, and receptor activity (48). Importantly, HMG contains both LH activity and additional FSH activity derived from urinary gonadotropins, whereas recombinant LH provides a more selective LH signal (49). Consequently, comparisons between the rFSH+rLH and rFSH+HMG groups should not be interpreted solely as differences in LH source. In clinical practice, adding HMG may alter the overall balance of exogenous gonadotropin exposure, as its FSH component can influence follicular recruitment and stimulation dynamics. Although the accompanying rFSH dose was generally adjusted according to ovarian response, residual differences in total FSH exposure may still have contributed to the observed variations in stimulation characteristics and reproductive outcomes. Thus, potential confounding from the mixed gonadotropin composition of HMG cannot be excluded. From a practical standpoint, recombinant LH may offer more selective LH activity and possibly lower gonadotropin exposure, whereas HMG is generally more accessible and less costly. The choice between these approaches should therefore be individualized based on patient characteristics, ovarian response, medication accessibility, and economic considerations.

Another potential explanation involves the distinct pharmacodynamic profiles of hCG-mediated LH activity and recombinant LH (47). Due to its longer half-life and higher receptor affinity compared to endogenous LH, hCG may induce excessive or prolonged LH receptor stimulation, potentially disrupting the delicate balance of FSH and LH signaling within the follicle (24, 49). Conversely, recombinant LH provides a more physiological pattern of receptor activation during the late follicular phase, supporting theca cell androgen production while preserving granulosa cell function (50). These mechanisms likely underlie the improved follicular development and embryo yield observed in the rFSH+rLH group, culminating in a higher CLBR.

Methodologically, appropriate handling of competing outcomes represents a key challenge in modelling CLBR. In a single ovarian stimulation cycle, live birth and permanent failure are mutually exclusive outcomes. Treating permanent failure as simple censoring in a Cox proportional hazards model may lead to overestimation of CLBR, as it implicitly assumes that future success remains possible. Competing risks methodology allows direct estimation of the CLBR while appropriately accounting for permanent failure as a competing event. Despite its methodological suitability, its application in IVF has been limited (51).

The present study has several notable strengths. First, the inclusion of a relatively large cohort of women of advanced reproductive age provided substantial statistical power to detect clinically meaningful differences between protocols. Second, the use of the CLBR as the primary endpoint is a major strength, as CLBR reflects the overall reproductive potential of a single stimulation cycle and is increasingly regarded as the most clinically relevant metric in ART research. Third, PSM was employed to balance baseline characteristics and minimize selection bias inherent to observational studies. Furthermore, the application of competing risk regression models enabled a more accurate evaluation of cumulative outcomes by accounting for permanent failure as a competing risk event.

Several limitations should be acknowledged. First, the retrospective, non-randomized design may introduce selection bias, particularly for the HMG group, the treatment allocation in clinical practice may reflect physician preference or perceived ovarian prognosis, factors that may not be fully captured despite PSM and multivariable adjustment. Second, PSM was performed separately for each pairwise comparison rather than within a unified three-group matched cohort, which limits direct statistical inference across all three groups simultaneously. Third, all patients were treated using a follicular-phase long-acting GnRH agonist protocol at a single center. Therefore, the external validity of the findings may be limited. Fourth, although the CLBR is a comprehensive efficacy measure, the absence of biological data, such as endocrine profiles or follicular fluid biomarkers, precludes mechanistic insights into the observed benefits of LH supplementation. In addition, because HMG contains both LH and FSH activity, the present comparisons cannot fully disentangle the specific effects of LH supplementation from differences in overall gonadotropin composition and exposure. Future prospective randomized trials are warranted to validate these results and further define the optimal candidates for recombinant LH supplementation.

## 5 Conclusions

In this retrospective cohort of women of advanced reproductive age undergoing ART, recombinant LH supplementation was associated with a modestly higher CLBR compared with rFSH alone. Although adjusted analyses suggested a potential association relative to rFSH+HMG, the absence of statistical significance in unadjusted analyses limits definitive interpretation. As an observational study, these findings represent associations rather than causal evidence. Prospective randomized controlled trials are needed to confirm these observations.

## Data Availability

The raw data supporting the conclusions of this article will be made available by the authors, without undue reservation.
